# Fabrication and Characterization of Porous Diopside/Akermanite Ceramics with Prospective Tissue Engineering Applications

**DOI:** 10.3390/ma16165548

**Published:** 2023-08-09

**Authors:** Adrian Ionut Nicoara, Andrada Elena Alecu, Gabriel-Costin Balaceanu, Eliza Maria Puscasu, Bogdan Stefan Vasile, Roxana Trusca

**Affiliations:** 1 Department of Science and Engineering of Oxide Materials and Nanomaterials, Faculty of Chemical Engineering and Biotechnologies, National University of Science and Technology Politehnica Bucharest, 011061 Bucharest, Romania; adrian.nicoara@upb.ro (A.I.N.); andrada.alecu16@gmail.com (A.E.A.); gabriel.balaceanu@stud.fim.upb.ro (G.-C.B.); truscaroxana@yahoo.com (R.T.); 2 National R&D Institute for Nonferrous and Rare Metals—IMNR, 077145 Bucharest, Romania; 3 National Research Center for Micro and Nanomaterials, National University of Science and Technology Politehnica Bucharest, 060042 Bucharest, Romania; bogdan.vasile@upb.ro; 4 Research Center for Advanced Materials, Products and Processes, National University of Science and Technology Politehnica Bucharest, 060042 Bucharest, Romania

**Keywords:** porous ceramic, hard tissue, diopside, akermanite, bone regeneration, sucrose

## Abstract

Tissue engineering requires new materials that can be used to replace damaged bone parts. Since hydroxyapatite, currently widely used, has low mechanical resistance, silicate ceramics can represent an alternative. The aim of this study was to obtain porous ceramics based on diopside (CaMgSi_2_O_6_) and akermanite (Ca_2_MgSi_2_O_7_) obtained at low sintering temperatures. The powder synthesized by the sol-gel method was pressed in the presence of a porogenic agent represented by commercial sucrose in order to create the desired porosity. The ceramic bodies obtained after sintering thermal treatment at 1050 °C and 1250 °C, respectively, were characterized by X-ray diffraction (XRD), scanning electron microscopy (SEM), and Fourier transform infrared spectroscopy (FTIR) to determine the chemical composition. The open porosity was situated between 32.5 and 34.6%, and the compressive strength had a maximum value of 11.4 MPa for the samples sintered at 1250 °C in the presence of a 20% wt porogenic agent. A cell viability above 70% and the rapid development of an apatitic phase layer make these materials good candidates for use in hard tissue engineering.

## 1. Introduction

Bone tissue has an increased capacity for regeneration, with certain mild conditions (cracks and small fractures) healing naturally over time. However, very serious lesions, with a surface of more than 2 cm, the so-called “critical-size defects,” cannot regenerate without help [1]. Traumatic injuries, degenerative diseases, histological diseases, congenital defects, or the removal of certain tumors can cause serious damage to bone tissue, requiring surgery to restore function and complete healing. From here derives the need for research and prioritization of the study of bone tissue regeneration through bone tissue engineering, with the main goal of overcoming the difficulties and limitations of traditional methods and increasing the efficiency of healing. Synthetic bone grafts can be proposed for fast bone regeneration. Some marketable synthetic biomaterials such as calcium phosphates [2], hydroxyapatite [3,4,5,6], and calcium sulphate [7] are known as natural bone replacers. The performance of these materials is a decisive factor for the precise reproduction of chemical parameters and morphological features in order to correlate with the biological behavior of the bone system.

Ceramic materials are the principal material used in the regeneration of bone tissue due to their having similar mechanical properties to bone: good hardness, low elasticity, tensile strength, compressive strength, Poisson’s ratio (lateral contraction), elongation percentage, etc. [8,9]. Ceramic materials perform very well due to their structural and chemical similarity to the mineral phase of native bone. In addition, ceramic stimulates bone differentiation and proliferation through osteogenic interactions. Ceramics have been used for filling, repairing bone defects, and surface coating materials for implants to improve integration in contact with the host bone. The best-known calcium phosphate ceramics are represented by HAp [5] and TCP (tricalcium phosphates) [10,11]. Bonding to bone is attributed to the formation of a layer of carbonated hydroxyapatite, similar to that in the bone phase [12,13].

However, the fragility and difficulty of modelling are still the most important limitations of these materials. That is why researchers have continued to develop and study different ceramic materials and combinations of ceramic materials that offer favorable results.

Some biomaterials used for hard tissue reconstruction are bioactive glass, calcium phosphates, and certain Ca and Mg silicates. Bioactive glass is made of materials based on amorphous silicates, which are compatible with the human body, are able to bind the bone and stimulate its growth, and have the ability to be resorbable over time [14,15]. During heat treatment at high temperature, bioglass usually crystallizes, favoring the development of biocompatible crystalline phases of diopside, akermanite, fluorapatite, and wollastonite [16]. These phases determine the strengthening of the scaffolding structure. Diopside (CaMgSi_2_O_6_) and akermanite (Ca_2_MgSi_2_O_7_) are calcium and magnesium silicates from the pyroxene class. Most of the properties of these ceramics are comparable to those of calcium phosphates (β-TCP, HAp), but the mechanical resistance is superior in most cases [17,18,19]. The good mechanical properties are mainly due to the microstructure and to the surfaces being provided with interconnected pores. Also, Ca^2+^, Mg^2+^, and Si^4+^ ions are nontoxic to the growth of osteoblasts and their differentiation, leading to apatite formation in both in vitro and in vivo studies [20,21].

The specialized literature proposes several methods of obtaining bioceramics, starting from the synthesis of diopside and/or akermanite powders produced by precipitation, synthesis in the solid phase, the sol-gel and solvothermal methods, etc. [17,18,19,22]. For example, in one study, akermanite synthesized by thermal treatment showed the presence of several phases even after calcination at 1300 °C for 3 h [23]. These ceramics have also been studied for use in hard-tissue engineering, but their properties still need to be adjusted to achieve a mechanical compressive strength comparable to that of bone tissue. For example, Chengtie Wu et al. [18] obtained, by using the polymer sponge method, pure diopside ceramics with open porosity between 75% and 95%. The mechanical compressive strengths of such scaffolds decrease from 1.360 MPa to 0.370 MPa. The relationship between compression strengths and porosity was also studied by Hamed Ghomi et al. [24]. In this study, Ghomi obtained at 1200 °C ceramic bodies based on diopside with an apparent porosity between 32 and 38%, for which he obtained mechanical resistances of 8.17 MPa and 5.37, respectively [24].

These values are higher than those for HAp ceramics, wollastonite, and 45S5 bioglass, but lower than those for natural bone [18,25]. For this reason, it is necessary to increase these mechanical strengths by reducing the open porosity and finding alternative routes to processing these ceramics. Venkatraman et al. [26] found that the use of high temperatures affects morphology, surface roughness, porosity, and particle size but also causes a decrease in bioactivity. Therefore, reducing the sintering temperatures of these ceramics is of interest.

The aim of this study was to obtain ceramics with adequate porosity that can be used in the regeneration of hard tissue. The proposed strategy was to lower the sintering temperature without sacrificing the compressive strengths. For this, two temperatures below 1300 °C were selected (1050 °C and 1250 °C, respectively). In order to induce porosity, 20 wt% sucrose was used. Thus, a dense but porous ceramic based on CaMgSi_2_O_6_ and Ca_2_MgSi_2_O_7_ was synthesized. This has been structurally, morphologically, and biologically characterized in order to use it as a bioactive material with potential in bone tissue reconstruction.

## 2. Materials and Methods

### 2.1. Powder Synthesis

**Figure 1:**
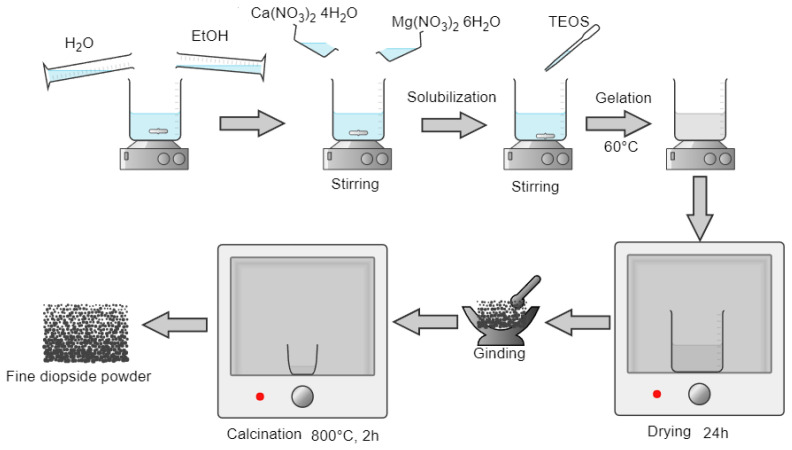
Schematic steps of diopside powder synthesis.

In this study, the synthesis of diopside powder was made starting from precursors such as Ca(NO_3_)_2_∙4H_2_O (Sigma-Aldrich, St. Louis, MO, USA, *p* ≥ 98%), Mg(NO_3_)_2_∙6H_2_O (Sigma-Aldrich, St. Louis, MO, USA, *p* ≥ 98%), (C_2_H_5_O)_4_Si (Sigma-Aldrich, St. Louis, MO, USA, reagent grade, *p* ≥ 98%), CH_3_CH_2_OH (Sigma-Aldrich, St. Louis, MO, USA, *p* ≥ 99.8%), and distilled water. The schematic steps for the sol-gel methods used are presented in Figure 1.

For 10 g of the final compound powder, the necessary quantities of precursors for maintaining the CaO:MgO:SiO_2_ molar ratio to 2:1:2 were wisely calculated and dosed.

The Ca(NO_3_)_2_∙4H_2_O and Mg(NO_3_)_2_∙6H_2_O were dissolved in 150 mL ethanol solution under magnetic stirring at room temperature. After 30 min of continuous stirring, the required amount of (C_2_H_5_O)_4_Si was added dropwise. Then the reaction mixture was kept at 60 °C on a hot plate to start the gelation process. The formed gel was matured for 24 h and then dried in an oven until all the liquid was eliminated, ground, and passed through a sieve with a mesh size below 100 microns. The obtained fine powder was calcinated at 800 °C for 2 h.

### 2.2. Obtaining the Ceramic Bodies 

**Figure 2:**
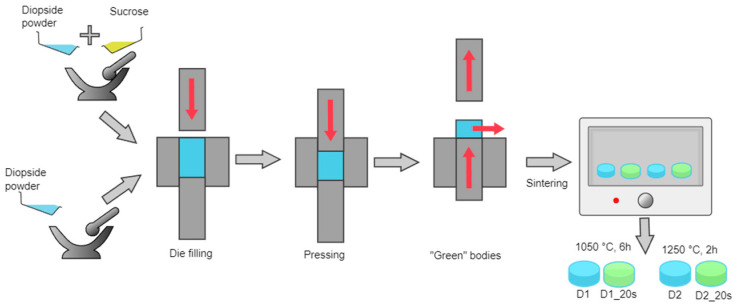
Schematic steps for obtaining ceramic bodies.

In order to obtain the ceramic bodies, the two strategies presented in Figure 2 were adopted:

- to obtain compact ceramic bodies, the powder previously obtained was pressed using a uniaxial press at 15 MPa in a die with a 13 mm diameter.
- to obtain a porous ceramic, the powder was mixed with 20 wt% commercial sucrose (Sigma-Aldrich, St. Louis, MO, USA, ≥99.5%) as a porogenic agent and pressed in the same conditions.

After pressing, the green bodies were sintered at 1050 °C for 6 h and at 1250 °C for 2 h; in all cases, the step of the thermal process was 10 °C/min. The maximum temperature used in this study (1250 °C) was selected because it was not necessary to approach the melting temperature of the diopside, which according to the literature is around 1391.5 °C [27].

The resulting sintered ceramic bodies can be found in Table 1.

The samples were coded according to the temperature and the presence of the porogenic agent. The samples sintered at 1050 °C were coded with D1, and those obtained at a higher temperature were coded with D2. The presence of the porogenic agent was highlighted by adding the code 20s to the name for the samples containing 20 wt% sucrose.

### 2.3. Characterization Methods

The structural information was obtained by the X-ray diffraction (XRD) technique, carried out in air, at room temperature, with the help of PANalytical Empyrean (Almelo, The Netherlands) equipment provided with a characteristic Cu X-ray tube (λ CuKα1 = 1.541874 Å). The samples were scanned in the 2θ angle range of 10–80° with a scan increment of 0.02° and a time of 100 s/step. The morphology of the obtained materials was evaluated by scanning electron microscopy (SEM), with a Quanta Inspect F50 microscope coupled with an energy dispersive spectrometer (EDS) (Thermo Fisher, Eindhoven, The Netherlands). The grain size distribution was determined by measuring the grain size using ImageJ 1.50i software (Wayne Rasband, National Institute of Health, 2016, Rockville, MD, USA). The mechanical compressive strength was determined with a speed of 1mm/min using Shimadzu Autograph AGS-X 20kN equipment (Shimadzu, Tokyo, Japan). The presence of characteristic chemical bonds was assessed by Fourier transform infrared spectroscopy (FT-IR) with a Nicolet iS50R spectrometer (Thermo Fisher, Waltham, MA, USA). The spectra were acquired at room temperature between 4000 and 400 cm^−1^ at a resolution of 4 cm^−1^ using the attenuated total reflection module (ATR).

The open porosity and absorption capacity of the obtained ceramic bodies were evaluated using methods described by Alecu et al. [20]. Initially, the samples were weighed in the air and then, after degassing under a vacuum for 15 min, immersed in a liquid of known density. Afterwards, the saturated samples were placed on a wet cotton cloth to remove the liquid from the surface and weighed again, both in the air and immersed in the liquid.

(1) $$\rho_{a}=\frac{w_{i}\;\times \;\rho_{x}}{w_{\mathrm{xa}}+{\;w}_{x}}\;(g/{\mathrm{cm}}^{3})$$

(2) $$A=\frac{w_{\mathrm{xa}}-w_{i}}{w_{\mathrm{xa}}}\;\times 100\;(\%)$$

(3) $$P=\frac{w_{\mathrm{xa}}-w_{i}}{w_{\mathrm{xa}}-w_{x}}\times 100\;(\%)$$

where w_i_—the initial weight of the sample (g); w_xa_—the weight (measured in air medium) of the liquid impregnated sample (g); w_x_—the weight (measured in ethanol medium) of the liquid impregnated sample (g); ρ_a_—apparent density (g/cm^3^); ρ_x_—water density (1 g/cm^3^); A—absorption capacity (%); and P—open porosity (%).

The volume shrinkage (C) after the heat treatment was calculated using Equation (4) by determining the height (h) and the diameter (d) of the cylindrical samples with the help of an electronic caliper. The determinations were made before and after the sintering process in order to calculate the shrinkage as volume (V) of the function. The weight loss (Δw) was determined using Equation (5) considering the weight of the sample before (w_0_) and after the sintering heat treatment (w_1_).

(4) $$C=\frac{\left(V_{f}-V_{i}\right)}{V_{i}}\times 100\;(\%)$$

(5) $$\Delta w=\frac{\left(w_{0}-w_{1}\right)}{w_{0}}\;\times 100\;(\%)$$

where: w_0_—the weight of the sample before sintering (g); w_1_—the weight of the sample after sintering (g); V_i_—the volume of the sample before sintering (cm^3^); V_f_—the volume of the sample after sintering (cm^3^).

Biomineralization capacity was evaluated by SEM coupled with EDS and FT-IR spectroscopy after immersing the ceramic bodies in SBF (simulated body fluid) solution at 37 °C for 14 days. The preparation of the SBF solution was performed by following Kokubo’s recipe and the standard procedure [28].

A cell viability assessment was performed using the LIVE/DEAD Viability/Cytotoxicity kit (Thermo Scientific, Waltham, MA, USA). The protocol involved culturing amniotic-fluid-derived stem cells (AFSCs—from the Coriell Institute (Kenton, NJ, USA)) under standard conditions of temperature and humidity (37 °C, 5% CO_2_, 90% humidity) and seeding in a 96-well plate, followed by the addition of the obtained materials. After 24 h of incubation, the ceramics were extracted and analyzed by fluorescence microscopy with a LSM 880 confocal microscope (Carl Zeiss, Göttingen, Germany). Calcein (Sigma-Aldrich, ≥95.0%, Darmstadt, Germany) was used to highlight the live cells (green fluorescence), and Ethidium Homodimer-1 (Sigma-Aldrich, Darmstadt, Germany) was used to highlight the dead cells (red fluorescence).

For the MTT evaluation, the samples were incubated for 24 h, followed by the addition of a mixture of 20 µL MTT (5 mg/mL) and 200 µL serum-free medium and 4 h incubation at 37 °C. The medium was completely removed, and dimethylsulfoxide-DMSO (200 µL) was added to each well in order to dissolve the formazan crystals resulting from the metabolic activity of the cells. The optical density was spectrophotometrically measured at 570 nm and 650 nm using the Beckman Coulter UV/VIS spectrophotometer, DU730 Molecular Devices (Beckman Coulter, Indianapolis, IN, USA).

For the analytical statistics, all the experiments were made in triplicate. The standard error of the mean and the statistical evaluation was performed using the Student’s *t*-test function, for which * *p* < 0.05, ** *p* < 0.01, respectively *** *p* < 0.001.

## 3. Results and Discussions

**Figure 3:**
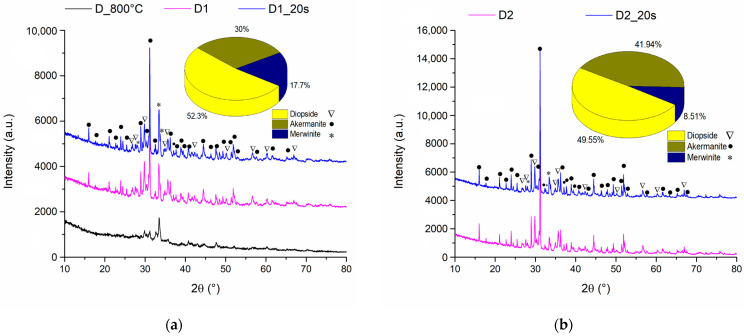
X-ray patterns of ceramic samples: untreated, treated at 1050 °C (**a**) in presence of sucrose (D1_20s), and without sucrose addition (D1) and treated at 1250 °C (**b**) in presence of sucrose (D2_20s) and without sucrose addition (D2).

Figure 3 represents the X-ray diffractogram for ceramic samples sintered at 1050 °C and 1250 °C with and without an added porogenic agent.

It is observed that at temperatures of 800 °C the diffraction interferences of the samples have a reduced intensity, which suggests a very low crystallinity degree. On the other hand, when the sample is calcined at 800 °C and then sintered at a temperature of 1050 °C, the diffraction peaks increase in intensity as a result of the crystallization of the interest compounds. This chemical composition was also confirmed by Choudhary et al. [29] in their study where by treating the precursors at a temperature of 800 °C for 6 h he obtained a mixture between the two phases of diopside and akermanite.

Rietveld analyses revealed an amount of 52.3% diopside (PDF 04-002-5549) and 30% akermanite (PDF 04-001-6874) but also the presence of the minority phase of 17.7% merwinite (PDF 04-011-6738).

When the sintering temperature was increased to 1250 °C (Figure 3b), an increase in the intensity of the diffraction maxima was observed, which indicates an increase in the crystallinity degree. Also, increasing temperature causes a decrease in the merwinite phase amount in accordance with the data reported by Duman and Bulut [30], the composition of the obtained ceramics being formed by diopside phases in the proportion of 49.55% and akermanite in the proportion of 41.94%.

The addition of the porogenic agent does not change the crystalline phase composition for any of the temperatures used.

**Figure 4:**
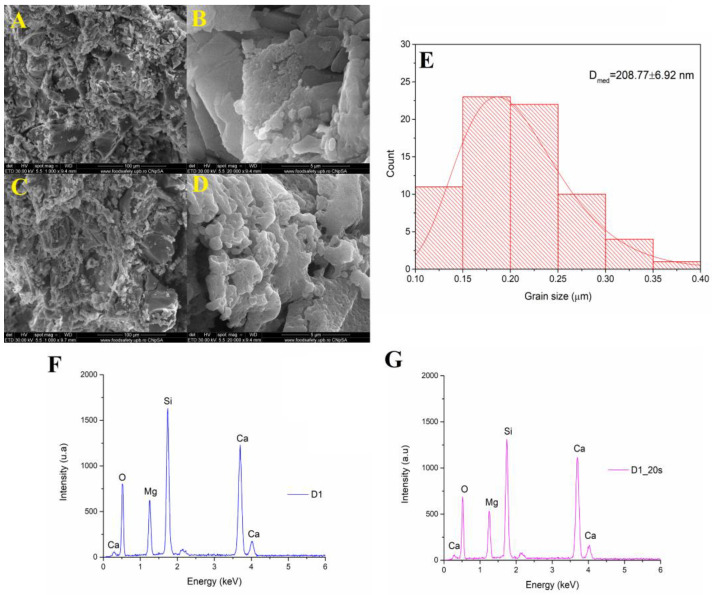
(**A**,**B**) SEM images magnification ×1000 and ×20,000 of D1 and (**C**,**D**) D1_20s samples; (**E**) grain size distribution of D1 sample; (**F**) EDS spectra for D1 and (**G**) D1_20s samples.

Figure 4 shows the SEM analyses of a sample treated at 1050 °C at a magnification of ×1000, respectively, ×20,000.

The microstructure consisted of well-sintered crystalline grains with a polyhedral shape and average dimensions of approximately 208 nm. Using a temperature of 1050 °C for 6 h determined the formation of porous surfaces even for the sample that did not have the additional addition of a porogenic agent. In the case of the ceramic sample marked D1_20s thermally treated in the presence of the porogenic agent, an increase in intergranular porosity could be observed.

The EDS spectra of samples D1 and D1_20s are shown in Figure 4F,G. Both the spectra indicate the presence of these characteristic elements: Ca, Mg, Si, and O. Thus, it is demonstrated that the sucrose did not remain in the composition of the material, being completely removed after the heat treatment.

**Figure 5:**
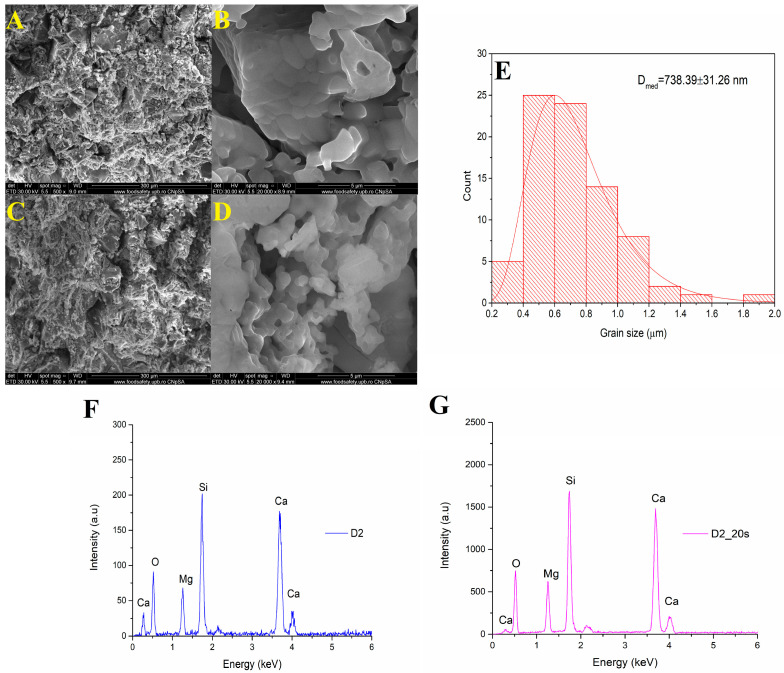
(**A**,**B**) SEM images magnification ×1000 and ×20,000 of D2 and (**C**,**D**) D2_20 samples; (**E**) grain size distribution of D2 sample and EDS spectra for D2 (**F**) and D2_20s (**G**) samples.

Figure 5 shows the SEM images of samples D2 and D2_20s at different magnifications. Well-sintered grains can be observed, with a predominantly spherical shape. Increasing the sintering temperature from 1050 °C to 1250 °C causes an increase in the size of the crystalline grains up to approximately 738 nm due to the coalescence and agglutination phenomenon, specific to the thermal sintering.

For the sample with added porogenic agent treated at 1250 °C, a higher frequency of pores present at the level of the structure could be observed compared to the sample without porogenic agent (D2). At higher magnifications (×20,000—see Figure 5D), an interconnected porous network caused by the removal of the sucrose by the thermal sintering treatment could be distinguished.

Figure 5F,G represents the EDS spectrum for samples D2 and D2_20s. The elements indicated following this analysis are: Ca, Mg, Si, and O. Thus, it is also demonstrated in the case of this sample that the sucrose did not intervene in changing the composition of the ceramic material.

**Figure 6:**
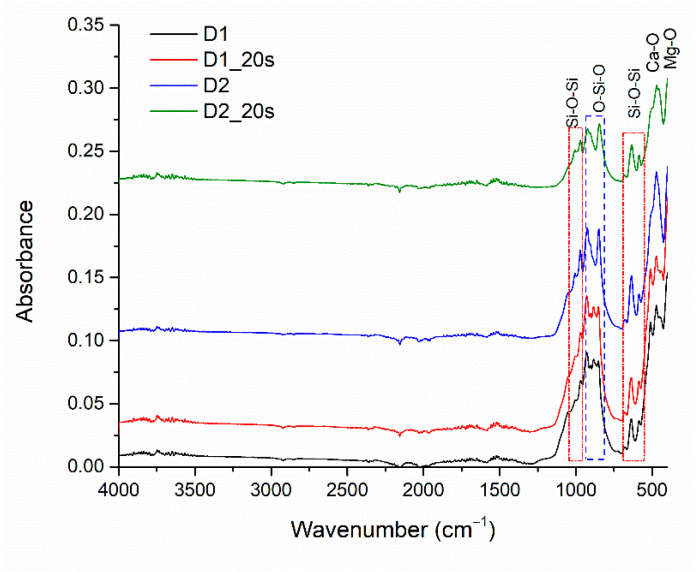
FT-IR Spectra for D1, D1_20s, D2, and D2_20s samples.

The absorption bands of the sintered samples are visible in Figure 6. The absorption bands located at 600 cm^−1^, 630 cm^−1^, 971 cm^−1^, and 1000 cm^−1^ correspond to the asymmetric stretching vibration of the Si-O-Si bond specific to the silica, in agreement with the literature [31]. The O-Si-O bond is highlighted by identifying absorption bands located around 850 cm^−1^ and 920 cm^−1^ [22]. Also, the presence of the Ca-O and Mg-O groups specific to the crystalline phases can be highlighted by the absorption bands at 420 cm^−1^ for Ca-O and 450 cm^−1^ for Mg-O [31,32]. FT-IR analysis demonstrates the results obtained from X-ray diffraction and EDS analysis.

**Figure 7:**
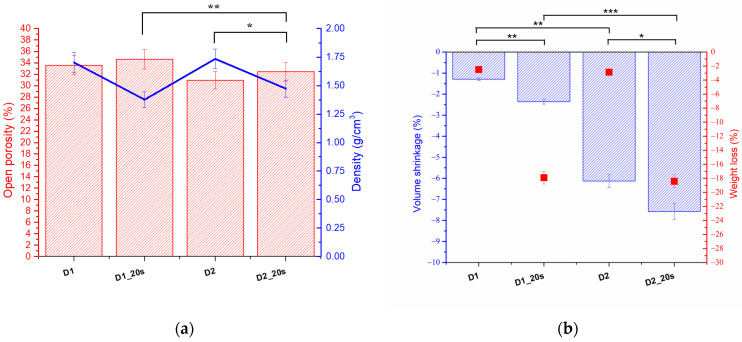
Porosity and density vs. temperature (**a**) and shrinkage and weight loss vs. temperature (**b**); * *p* < 0.05, ** *p* < 0.01, respectively *** *p* < 0.001.

The open porosity, density, shrinkage, and weight loss were evaluated according to the method proposed by Alecu at al. [20] using Equations (1)–(5). The values obtained for samples D1, D1_20s, D2, and D2_20s are shown in Figure 7.

The samples in which the porogenic agent was introduced had a higher porosity than the samples without the porogenic agent. The best porosity, of approximately 34.6%, was obtained for sample D1_20s, followed by sample D1, with 33.5%.

With increasing temperature, from 1050 °C to 1250 °C (D2 and D2_20s), a slight effect of decreasing porosity was observed. This can be explained by the fact that once the temperature increases, the degree of sintering increases as a result of agglutination. Following this process, the pores decrease in size and the sample tends to compact.

The samples with the porogenic agent (D2_20s) present a porosity of 32.5%, higher than the sample without the porogenic agent (D2), which has a value of 30%. The porosity obtained for this type of material falls within the range of cortical bone tissue [33,34]. It can be seen from the analysis that the apparent densities approach the bone tissue range.

Figure 7b shows the volume shrinkage and the mass loss related to the treatment temperatures of the samples.

As expected, sample D2_20s, which contained sucrose and was treated at the highest temperature, had the highest mass loss and the highest volume shrinkage. This aspect suggests that, following the heat treatment, the porogenic agent was removed from the ceramic structure. In the case of the samples sintered at 1050 °C, which were subjected to a lower heat treatment, the volume shrinkage was 1.29% for sample D1 and 2.35% for sample D2_20s. This analysis is consistent with the XRD and FTIR analyses, which showed that the porogenic agent was removed upon heat treatment.

**Figure 8:**
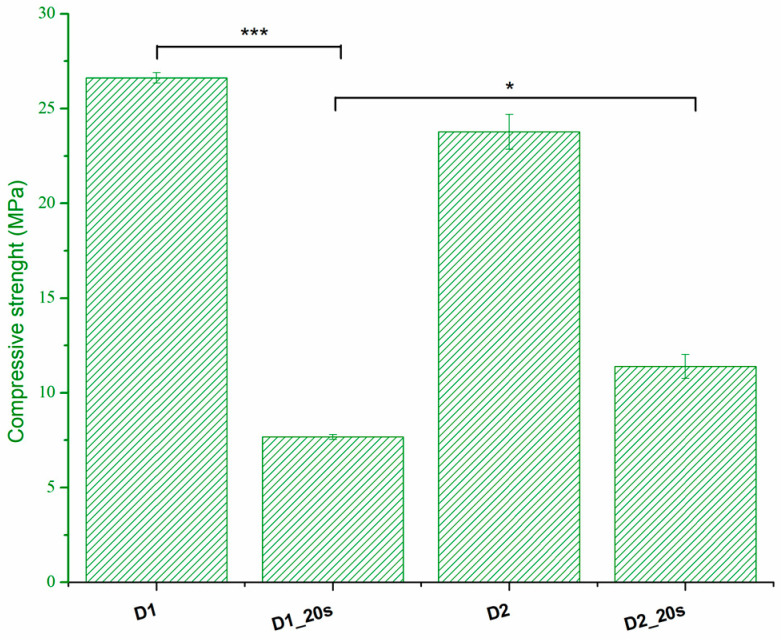
The compression strength of all ceramic samples; * *p* < 0.05, *** *p* < 0.001.

The compressive strength is presented in Figure 8.

The thermal treatment at 1050 °C for a long period of time (6 h) determines a good sintering of the samples, which leads to an increase in compressive strength. Thus, sample D1 presents the highest compressive strength with a value of 26.6 MPa. The addition of the porogenic agent induces a decrease in the compressive strength up to a maximum of 7.6 MPa. This behavior is in agreement with the data presented by Ghomi et al. [24] and can be attributed to the increase in apparent porosity. Increasing the sintering temperature to 1250 °C causes an increase in compression strength as a result of the densification phenomenon. This can be observed in the case of samples with added porogenic agents. When the porosity decreases from 34.6% for sample D1_20s to 32.5% for sample D2_20s, the strength increases from 7.6 MPa to 11.4 MPa. The strengths reported in the present study are higher than those reported in the literature for similar porosities and are found in the range of trabecular bone tissue, 0.1–16 MPa [24,25].

The first test used to evaluate the biocompatibility of the materials obtained was the immersion of the samples in the SBF.

**Figure 9:**
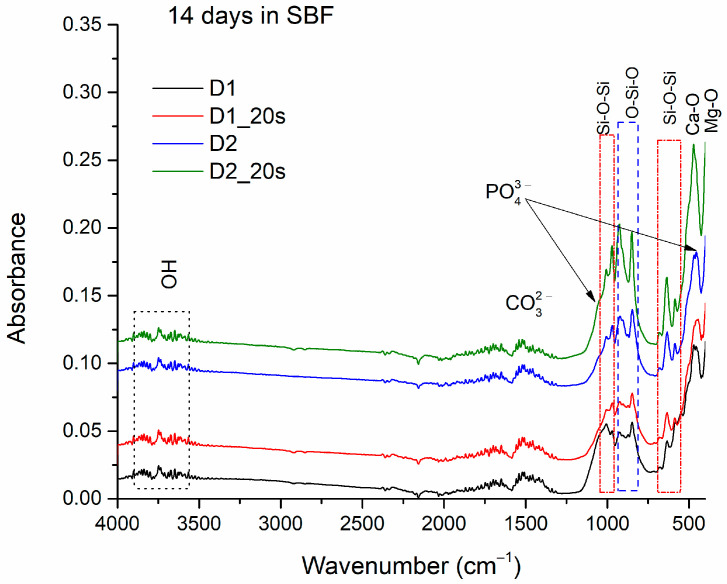
FTIR spectra for samples D1, D1_20s, D2, D2_20s after 14 days in SBF.

Figure 9 shows the absorption band of the apatite phase clusters adhering to samples D1, D1_20s, D2, and D2_20s after immersion for 14 days in the SBF.

In addition to the main bonds within the samples, specified previously in the FTIR analysis of the materials (see Figure 6), the presence of O-H bonds can also be observed, at specific stretching vibrations at 3505–3902 cm^−1^ and in the range of 1650–1850 cm^−1^ [3].

Also, the CO_3_^2−^ ion is identified with the stretching vibrations found in the range of 1508–1551 cm^−1^, suggesting the formation of a carbonated hydroxyapatite, also known as “B” type apatite or biological apatite [24,35]. The absorption bands at 1010 cm^−1^ and 470 cm^−1^ can be associated with the PO_4_^2−^ ion [3,18,36].

**Figure 10:**
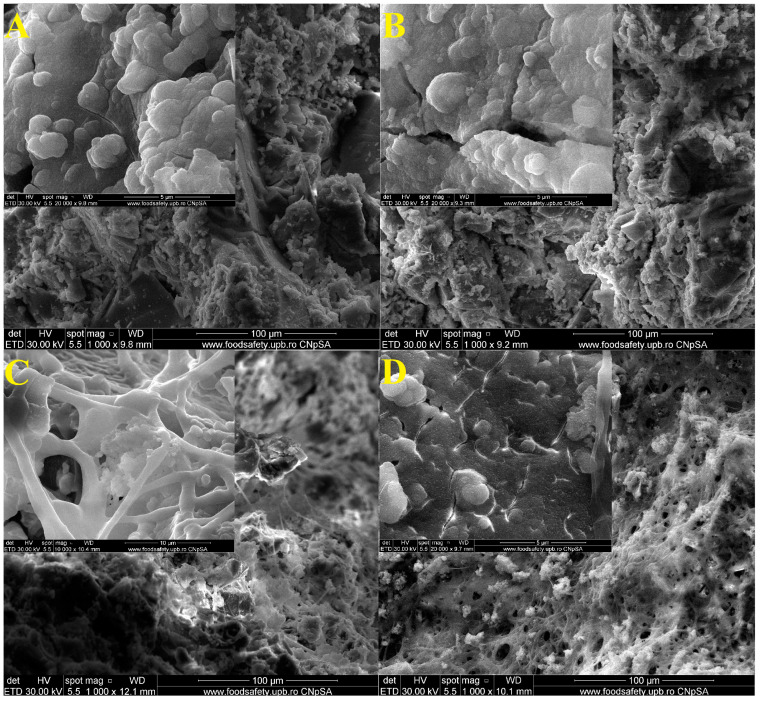
SEM images of samples D1 (**A**) and D1_20s (**B**), D2 (**C**) and D2_20s (**D**) after 14 days in SBF.

Figure 10 shows the SEM images of samples D1, D1_20s, D2, and D2_20s after 14 days in the SBF.

In the SEM images, the deposits of some apatite phase particles are highlighted. This is also suggested by the decrease in the pores of the samples, meaning that those particles characteristic of the biological apatite phase managed to penetrate inside them. Thus, the surface of the materials became mineralized [18].The rounded morphology particles could be attributed, according Yamagata et al. [37], to the formation of hydroxyapatite on the sample surface.

**Figure 11:**
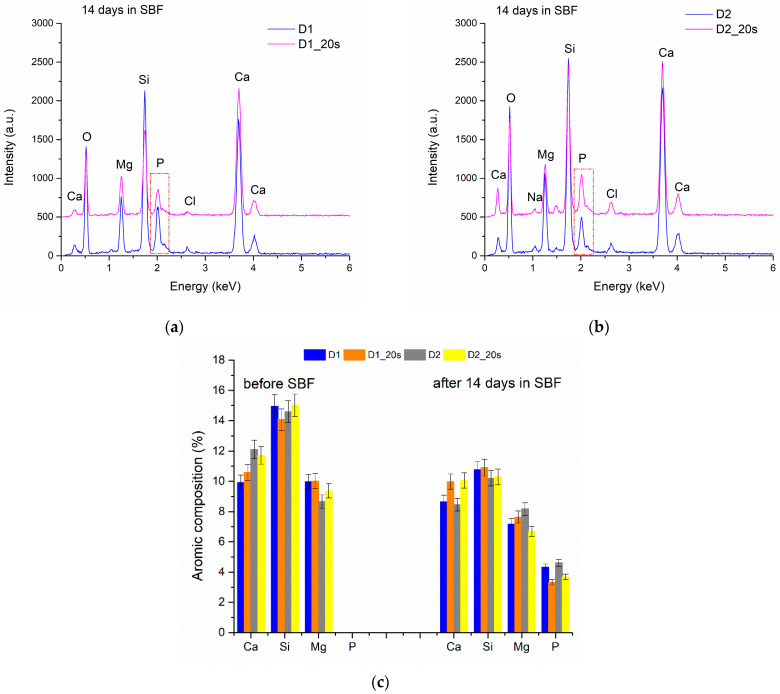
EDS spectra for samples D1, D1_20s (**a**) and D2, D2_20s (**b**) after 14 days in SBF; (**c**) atomic composition determined by EDS before and after 14 days in SBF.

The EDS spectra performed on all the samples (Figure 11) indicate the presence of Ca and P (3.33–4.61 at%), characteristic of the early formation of the apatite phase on the ceramic surface.

Immersion in SBF for 14 days causes a decrease in the element’s atomic concentration in the ceramic bodies as a result of dissolution (Figure 11c). All the studied ceramics showed a similar behavior. After 14 days, the amount of Si decreased by approximately 4.4%. These results are comparable with data in the literature showing that the release of Si ions is proportional to the decrease in the sample weight and to the immersion time in the SBF [18,19,37].

Thus, the presence of these types of bonds demonstrates the formation of an apathetic phase on the surface of all the samples, thus proving their bioactive character.

**Figure 12:**
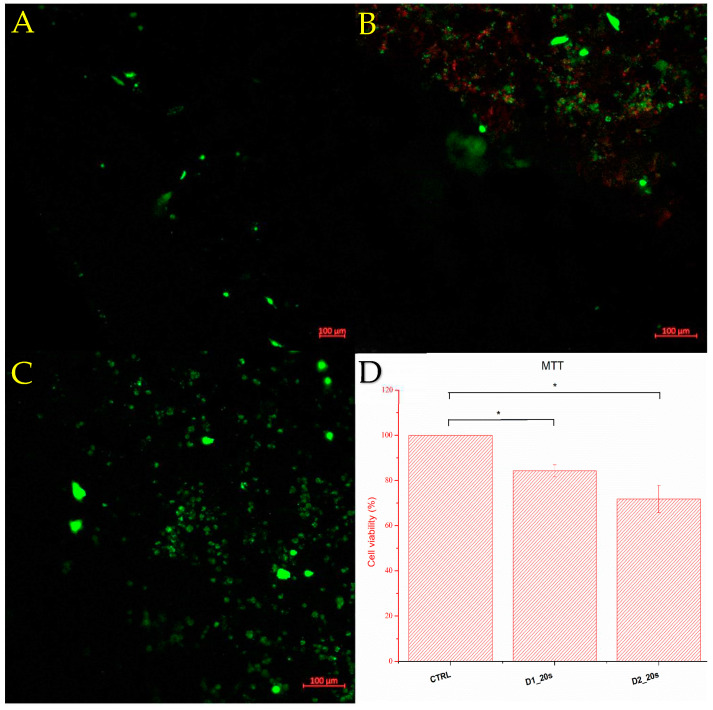
Florescence microscopy for images for D1_20s (**A**) and D2_20s (**B**) control (**C**) samples and cell viability (MTT) (**D**) after 7 days (green fluorescence—living cells; red color—dead cells); * *p* < 0.05.

Figure 12 shows the cell viability determined by the MTT test and fluorescence microscopy of the ceramic samples containing the porogenic agent, namely D1_20s and D2_20s.

Viability was measured seven days after seeding the samples with the culture medium. Sample D1_20s clearly showed good viability, around 84.25%. This means that the sample is biocompatible and does not exhibit cytotoxicity for the amniotic-fluid-derived stem cells. Also considering the good resistance to compression, as well as a porosity close to the trabecular bone standard, it can be stated that the material synthesized within sample D1_20s is suitable for this type of application, showing potential in the healing of trabecular bone tissue conditions. Following the behavior of sample D2_20s, it can be observed from the cell viability of 71.76% that the material has a more-than-decent value.

It is observed that the cells adhered to the surface of the porous ceramic. According to the MTT test, cells adhere better on the surface of porous ceramics treated at a temperature of 1050 °C. Increasing the temperature to 1250 °C decreases cell viability, with a larger number of dead cells (seen in red) observed on the fluorescence image. This temperature-induced behavior is consistent with the data reported by Venkatraman et al. [26] which showed a decrease in cell viability with increasing sintering temperature.

Following this analysis, it can be concluded that the samples containing a mixture of phases, diopside, and akermanite do not present a danger for a biological tissue that could adhere to the substrate of a construct made of these ceramics. The biocompatibility of these materials can still be improved by functionalizing the surface with certain factors to modulate the cell behavior: bone morphogenic proteins, growth factors, or antibodies [38].

Since the obtained ceramics have a supporting role until the formation of the new bone occurs, the mechanical behavior must also be evaluated after immersion in the simulated body fluids. Although the ceramics obtained in our study have high open porosity, the pore structure and shape must be modelled to create scaffolds with interconnected porosity. According to Ahamadipour et al. [39], interconnected pores help cells proliferate. However, porosity needs to be increased without scarifying mechanical strength. The additive manufacturing (3D printing) of scaffolds based on diopside and akermanite could represent a solution.

Also, in future studies, the properties of osteoinduction and osteoconduction should be evaluated, as should the rate of release of ions from the ceramic composition.

## 4. Conclusions

In this study, starting from the sol-gel method, diposide and akermanite ceramic were obtained and characterized. The phase characterization showed a poor phase crystallization at 800 °C, but at high temperatures (1050 °C respectively 1250 °C), two majority crystalline phases consisting of diopside and akermanite were formed. This merwinitic phase (identified as a minority-formed phase) decreased in quantity as the sintering temperature increased. The addition of a porogenic agent led to obtaining ceramic materials with controlled porosity situated between 32 and 35%. A compressive strength comparable to natural trabecular bone in in vitro tests showed good mineralization capacity in the porous ceramic surfaces, determining the formation of a consistent layer of hydroxyapatite which stimulates cell growth. The use of higher sintering temperatures tends to decrease the biological activity of these types of ceramics.

## Figures and Tables

**Table 1:** Sample formulation and thermal conditions for obtaining ceramic bodies.

Sample Name	Sintering Temperature	Time	Porogenic Agent (wt%)
D1	1050 °C	6 h	0%
D2	1250 °C	2 h	
D1_20s	1050 °C	6 h	20%
D2_20s	1250 °C	2 h	

## Data Availability

Not applicable.
